# Supplementary material for: Extreme environments simplify reassembly of communities of arbuscular mycorrhizal fungi
Source: mSystems. 2024 Feb 20;9(3):e01331-23. doi: 10.1128/msystems.01331-23 (PMC10949450; doi:10.1128/msystems.01331-23)
Supplement: Supplemental Material — Supplemental tables and figures. [file msystems.01331-23-s0001.docx]

**Supplemental Material**

**Extreme Environments Simplify Reassembly of Communities of Arbuscular Mycorrhizal Fung****i**

**Nataša Šibanc^a,b^, Dave R. Clark^c, d^, Thorunn Helgason^e, f^, Alex J. Dumbrell^c,^*, Irena Maček^g,^***

Department of Agronomy, Biotechnical Faculty, University of Ljubljana, Ljubljana, Slovenia^a^ present address: Slovenian Forestry Institute, Ljubljana, Slovenia^b^; School of Life Sciences, University of Essex, Colchester United Kingdom^c^; Institute for Analytics and Data Science, University of Essex, Colchester, United Kingdom^d^; Department of Biology, University of York, York, United Kingdom^e^ present address: Institute for Ecology and Evolution, School of Biological Sciences, University of Edinburgh, Scotland^f^, Department of Biology, Biotechnical Faculty, University of Ljubljana, Ljubljana, Slovenia^g^

**Corresponding authors:**

*Dr Irena Maček (irena.macek@bf.uni-lj.si), Department of Biology, Biotechnical Faculty, University of Ljubljana, Večna pot 111, 1000 Ljubljana, Slovenia. Tel: +386 (0)1320 3343.

*Prof. Alex J. Dumbrell (adumb@essex.ac.uk), School of Life Sciences, University of Essex, Colchester, United Kingdom. Tel. +44 (0)1206 87 2539.

**TABLE S1.** Botanical survey conducted at Slovenian meadow sites. Plant species, found only at one area (high CO_2_ exposed or control), are indicated in bold.

| High CO_2_ exposed | Control |
| --- | --- |
| *Achillea millefolium* L. | *Achillea millefolium* L*.* |
| *Agrostis canina* L. | *Agrostis canina* L. |
| *Agrostis stolonifera* L. | *Agrostis stolonifera* L. |
| ***Betonica officinalis* L.** | ***Ajuga reptans* L.** |
| *Carex hirta* L. | *Carex hirta* L. |
| *Centaurea jacea* L. | *Centaurea jacea* L. |
| *Erigeron annuus* (L.) Pers*.* | ***Cerastium glomeratum* Thuill.** |
| *Juncus conglomeratus* L. | ***Convolvulus arvensis* L.** |
| *Juncus effusus* L. | ***Dactylis glomerata* L.** |
| ***Leontodon autumnalis* L.** | ***Echinochloa crus-galli* (L.) PB** |
| *Leontodon hispidus* L. | *Erigeron annuus* (L.) Pers*.* |
| *Lotus corniculatus* L. | ***Galium verum* L.** |
| ***Lythrum salicaria* L.** | ***Holcus lanatus* L.** |
| *Plantago lanceolata* L. | *Juncus conglomeratus* L. |
| ***Poa annua* L.** | *Juncus effusus* L. |
| *Poa pratensis* L. | ***Lathyrus pratensis* L.** |
| ***Polygonum arenastrum* Boreau** | *Leonthodon hispidus* L. |
| ***Polygonum aviculare* L.** | ***Leucanthemum ircutianum* (Turcz.) DC.** |
| *Ranunculus repens* L. | *Lotus corniculatus* L. |
| *Scirpus sylvaticus* L. | ***Lycopus europaeus* L.** |
| *Solidago gigantea* Aiton | ***Lysimachia nummularia* L.** |
| *Taraxacum officinale* Weber in Wiggers | ***Lysimachia vulgaris* L.** |
| *Trifolium pratense* L. | *Plantago lanceolata* L. |
| *Trifolium repens* L. | *Poa pratensis* L. |
| ***Veronica officinalis* L.** | *Ranunculus repens* L. |
| ***Vicia* sp.** | *Scirpus sylvaticus* L. |
|  | *Solidago gigantea* Aiton |
|  | ***Stellaria media (L.)* Vill.** |
|  | ***Tanacetum vulgare* L.** |
|  | *Taraxacum officinale* Weber in Wiggers |
|  | *Trifolium pratense* L. |
|  | *Trifolium repens* L. |
|  | ***Veronica acinifolia* L.** |
|  | ***Veronica persica* Poir.** |
|  |  |

Total richness:

26 plant species 34 plant species

**TABLE S2.** Soil characteristics data of the high CO_2_ exposed and control soils for Slovenian meadow sites, used in this study: soil pH, available phosphorus (P_2_O_5_), total nitrogen (N), % (v/v) carbon dioxide (CO_2_) and % (v/v) oxygen (O_2_) in soil air. Values are mean ± standard deviation (*n* = 3).

|  |  | pH | P_2_O_5_ | N total | CO_2_ | O_2_ |
| --- | --- | --- | --- | --- | --- | --- |
| 2010 | |  | mg/100g | % | % | % |
| April | Control | 4.8 ± 0.5 | 7.6 ± 4.6 | 0.32 ± 0.05 | 0.9 ± 0.5 | 20.0 ± 0.2 |
|  | High CO_2_ | 4.3 ± 0.2 | 11.6 ± 5.4 | 0.35 ± 0.06 | 59.2 ± 18.2 | 8.2 ± 3.0 |
| June | Control | 4.7 ± 0.5 | 7.6 ± 3.5 | 0.32 ± 0.06 | 1.1 ± 1.0 | 19.2 ± 0.7 |
|  | High CO_2_ | 4.2 ± 0.1 | 12.2 ± 5.3 | 0.37 ± 0.09 | 61.8 ± 11.6 | 7.0 ± 1.8 |
| August | Control | 5.0 ± 0.8 | 7.8 ± 5.3 | 0.29 ± 0.06 | 0.6 ± 0.3 | 19.4 ± 0.2 |
|  | High CO_2_ | 4.2 ± 0.2 | 14.5 ± 6.6 | 0.35 ± 0.08 | 67.3 ± 15.3 | 7.7 ± 2.3 |
| October | Control | 4.9 ± 0.7 | 9.2 ± 4.2 | 0.30 ± 0.07 | 0.6 ± 0.3 | 20.3 ± 0.4 |
|  | High CO_2_ | 4.2 ± 0.2 | 14.4 ± 6.6 | 0.33 ± 0.06 | 75.7 ± 19.4 | 7.5 ± 5.0 |
| 2011 | |  |  |  |  |  |
| April | Control | 4.8 ± 0.4 | 8.2 ± 6.0 | 0.31 ± 0.08 | 1.0 ± 0.6 | 19.8 ± 0.5 |
|  | High CO_2_ | 4.2 ± 0.2 | 11.7 ± 5.5 | 0.33 ± 0.05 | 64.2 ± 11.9 | 9.6 ± 3.0 |
| June | Control | 4.7 ± 0.5 | 6.1 ± 3.7 | 0.30 ± 0.07 | 1.6 ± 0.7 | 18.8 ± 0.6 |
|  | High CO_2_ | 4.2 ± 0.2 | 12.3 ± 5.0 | 0.35 ± 0.04 | 73.2 ± 14.9 | 7.0 ± 2.7 |
| August | Control | 4.8 ± 0.4 | 8.3 ± 4.3 | 0.33 ± 0.09 | 0.5 ± 0.3 | 19.3 ± 0.6 |
|  | High CO_2_ | 4.0 ± 0.2 | 17.8 ± 10.5 | 0.41 ± 0.04 | 68.5 ± 17.2 | 7.2 ± 3.5 |
| October | Control | 4.6 ± 0.6 | 8.7 ± 4.1 | 0.31 ± 0.05 | 0.6 ± 0.6 | 20.7 ± 0.2 |
|  | High CO_2_ | 4.0 ± 0.2 | 16.3 ± 6.9 | 0.34 ± 0.06 | 73.2 ± 14.3 | 10.0 ± 3.4 |

**TABLE S3.** Soil characteristics data of high CO_2_ exposed and control soils for the Slovenian forest sites, used in the study of the impact of abiotic environmental factors on biodiversity of AM fungi: soil pH, available phosphorus (P_2_O_5_), total nitrogen (N), % (v/v) carbon dioxide (CO_2_) and % (v/v) oxygen (O_2_) in soil air. Values are means ± standard deviation (*n* = 4). Significant differences between high CO_2_ exposed and control samples are indicated in boldface (*P* < 0.05; Kruskal-Wallis rank sum test).

|  |  | **pH** | **P_2_O_5_** | **Total N** | **CO_2_** | **O_2_** |
| --- | --- | --- | --- | --- | --- | --- |
| 2010 | |  | **mg/100g** | **%** | **%** | **%** |
| April | Control | 3.5 | 5.5 | 0.38 | **0.4 ± 0.1** | **20.6 ± 0.1** |
|  | High CO_2_ | 3.4 | 36.9 | 0.92 | **91.9 ± 5.8** | **3.3 ± 1.7** |
| June | Control | 3.5 | 5.8 | 0.34 | **0.5 ± 0.3** | **19.8 ± 0.4** |
|  | High CO_2_ | 3.5 | 42.5 | 0.71 | **89.5 ± 8.4** | **3.1 ± 1.4** |
| August | Control | 3.4 | 9.1 | 0.51 | **0.8 ± 0.2** | **20.1 ± 0.1** |
|  | High CO_2_ | 3.7 | 96.8 | 0.44 | **78.6 ± 22.1** | **5.1 ± 3.9** |
| October | Control | 3.6 | 5.4 | 0.31 | **1.0 ± 0.1** | **20.3 ± 0.1** |
|  | High CO_2_ | 3.7 | 76.3 | 0.61 | **97.0 ± 3.7** | **4.8 ± 2.3** |
| 2011 | |  |  |  |  |  |
| April | Control | 3.3 | 3.6 | 0.44 | **0.5 ± 0.2** | **20.3 ± 0.1** |
|  | High CO_2_ | 3.1 | 12.8 | 0.68 | **72.5 ± 4.3** | **12.5 ± 0.9** |
| June | Control | 3.5 | 5.8 | 0.85 | **1.2 ± 0.3** | **20.3 ± 0.2** |
|  | High CO_2_ | 3.4 | 33.4 | 1.33 | **85.8 ± 12.9** | **6.5 ± 4.1** |
| August | Control | 3.3 | 6.0 | 0.55 | **0.5 ± 0.1** | **20.0 ± 0.2** |
|  | High CO_2_ | 3.2 | 96.8 | 0.39 | **91.4 ± 12.4** | **3.0 ± 2.0** |
| October | Control | 3.2 | 4.2 | 0.51 | **0.6 ± 0.2** | **20.6 ± 0.2** |
|  | High CO_2_ | 3.5 | 41.4 | 0.99 | **77.4 ± 5.1** | **12.5 ± 1.3** |

**TABLE S4.** Soil characteristics data of high CO_2_ exposed and control soils for the Czech sites, used in the study of the impact of abiotic environmental factors on biodiversity of AM fungi: soil pH, available phosphorus (P_2_O_5_), total nitrogen (N), % (v/v) carbon dioxide (CO_2_) and % (v/v) oxygen (O_2_) in soil air. Values are means ± standard deviation (*n* = 6 replicates for control samples and *n* = 8 replicates for high CO_2_ exposed samples). Significant differences between high CO_2_ exposed and control samples are indicated in boldface (*P* < 0.05; Kruskal-Wallis rank sum test).

|  | **pH** | **P_2_O_5_** | **Total N** | | **CO_2_** | | **O_2_** | |  |
| --- | --- | --- | --- | --- | --- | --- | --- | --- | --- |
|  | **v CaCl_2_** | **mg/100g** | | **%** | | **%** | | **%** | |
| Control | **4.4 ± 0.4** | **9.7 ± 2.3** | **0.44 ± 0.17** | | **0.8 ± 0.6** | | **18.8 ± 1.5** | |  |
| High CO_2_ | **3.9 ± 0.2** | **14.6 ± 5.1** | **1.15 ± 0.26** | | **78.7 ± 20.7** | | **5.5 ± 4.4** | |  |

**TABLE S5.** Soil characteristics data of high CO_2_ exposed and control soils for the Italian sites, used in the study of the impact of abiotic environmental factors on community structure of AM fungi: soil pH, available phosphorus (P_2_O_5_), total nitrogen (N) and flux (µmol CO_2_ m^-2^ s^-1^) of carbon dioxide (CO_2_). Values are means ± standard deviation (high CO_2_ exposed: *n* = 3 for *Agrostis stolonifera, n* = 4 for *Phragmites australis, n* = 4 for *Plantago lanceolata,* *n* = 3 for *Centaurea alba*; control samples: *n* = 4 for *Agrostis stolonifera, n* = 2 for *Phragmites australis, n* = 3 for *Plantago lanceolata,* *n* = 3 for *Centaurea alba*). Significant differences between high CO_2_ exposed and control samples are indicated in boldface (*P* < 0.05; Kruskal-Wallis rank sum test).

| **Rhizosphere soil** | | **pH** | | **P_2_O_5_** | | **Total N** | | **CO_2_** | |
| --- | --- | --- | --- | --- | --- | --- | --- | --- | --- |
|  | | **v CaCl_2_** | | **mg / 100 g** | | **%** | | **µmol CO_2_ m^-2^ s^-1^.** | |
| ***Agrostis stolonifera*** |  | |  | |  | |  | |  |
| Control | 6.5 ± 0.2 | | 24.6 ± 11.8 | | 2.19 ± 0.10 | | **16.05 ± 2.32** | |  |
| High CO_2_ | 6.5 ± 0.1 | | 32.7 ± 15.0 | | 1.73 ± 0.65 | | **171.56 ± 59.89** | |  |
| ***Phragmites australis*** | | | | | | | | |  |
| Control | 6.6 ± 0.3 | | 55.7 | | 1.00 ± 0.15 | | **21.93 ± 5.64** | |  |
| High CO_2_ | 4.2 ± 0.8 | | 107.5 ± 14.9 | | 0.99 ± 0.42 | | **269.83 ± 49.44** | |  |
| ***Plantago lanceolata*** |  | |  | |  | |  | |  |
| Control | 7.0 ± 0.2 | | 32.0 ± 14.2 | | 1.32 ± 0.32 | | **15.10 ± 8.33** | |  |
| Medium CO_2_ | 6.7 ± 0.3 | | 19.4 ± 17.1 | | 1.33 ± 0.52 | | **40.70 ± 7.74** | |  |
| ***Centaurea alba*** |  | |  | |  | |  | |  |
| Control | **7.0 ± 0.2** | | 32.0 ± 14.2 | | 1.32 ± 0.32 | | **20.50 ± 3.54** | |  |
| High CO_2_ | **6.5 ± 0.1** | | 32.7 ± 15.0 | | 1.73 ± 0.65 | | **169.72 ± 55.96** | |  |

**TABLE S6.** The height of plants measured from soil cores taken at Slovenian meadow sites for analysis of AM fungal community composition.

| Sampling | Gas regime | Mean ± SD | Kruskal-Wallis rank sum test |
| --- | --- | --- | --- |
| April 2010 | High CO_2_ | 8.00 ± 5.50 | chi-squared = 116.35, df = 1, P < 0.001 |
|  | Control | 15.49 ± 5.88 |  |
| June 2010 | High CO_2_ | 10.30 ± 5.04 | chi-squared = 88.16, df = 1, P < 0.001 |
|  | Control | 21.33 ± 8.93 |  |
| August 2010 | High CO_2_ | 11.33 ± 7.16 | chi-squared = 33.17, df = 1, P < 0.001 |
|  | Control | 19.18 ± 9.08 |  |
| October 2010* |  |  |  |
|  |  |  |  |

* In October 2010, the meadow was mown.

**TABLE S7.** Climate variables for the Slovenian meadow and forest sites, obtained from Gornja Radgona weather station. Presented is the mean (± SD) of 30 days, separately for maximal temperature, minimal temperature and sun hours, finishing on the last day of each sampling (4 May, 30 June, 18 August, 29 October 2010 and 20 April, 29 June, 26 August, 26 October 2011) and sum of rainfall for each 30-day period.

| Sampling | T maximal (°C) | T minimal (°C) | Sun (h) | Rain (mm) |
| --- | --- | --- | --- | --- |
| April 2010 | 17.8 ± 5.0 | 6.2 ± 3.7 | 6.2 ± 4.4 | 36.8 |
| June 2010 | 25.0 ± 5.2 | 13.7 ± 2.9 | 7.9 ± 5.8 | 86.4 |
| August 2010 | 26.4 ± 3.2 | 15.0 ± 1.7 | 7.1 ± 3.9 | 123.4 |
| October 2010 | 13.3 ± 2.9 | 4.0 ± 4.3 | 3.1 ± 3.1 | 46.7 |
| April 2011 | 18.6 ± 3.9 | 3.5 ± 2.9 | 7.2 ± 3.6 | 19.6 |
| June 2011 | 25.9 ± 2.6 | 13.6 ± 2.2 | 8.8 ± 4.2 | 88.0 |
| August 2011 | 27.9 ± 3.7 | 15.2 ± 2.2 | 8.9 ± 3.8 | 97.6 |
| October 2011 | 17.7 ± 6.6 | 4.8 ± 4.4 | 6.0 ± 4.2 | 84.2 |


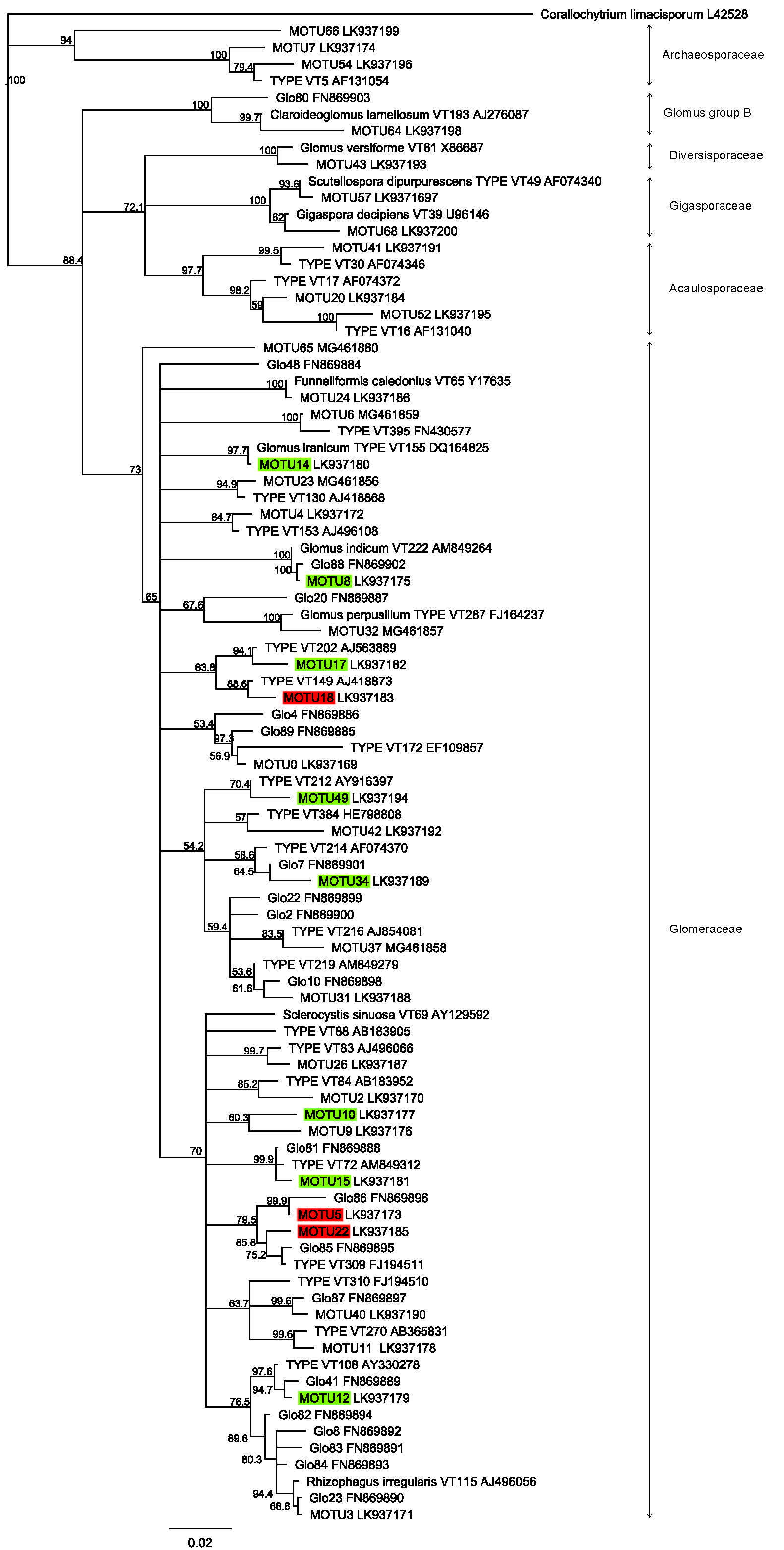


**FIG S1.** Neighbor-joining phylogeny of AM fungi MOTU recorded at Slovenian meadow and forest sites, Italian and Czech sites, their closest virtual taxa (VT, MaarjAM database, 1) and representative sequences of AM fungi from Slovenian meadow sites sampled in 2007 (Glo, 2). Bootstrap values (10,000 replicates) are shown above the branches and before the node to which they correspond. Green colour is representing MOTUs with the significantly higher abundance of sequences from control area, and red colour is representing MOTU with the significantly higher abundance of sequences from mofette area (GLM). The tree is rooted with Corallochytrium limacisporum as a general outgroup to all fungi.

1. Öpik M, Vanatoa A, Vanatoa E, Moora M, Davison J, Kalwij JM, Reier Ü, Zobel M. 2010. The online database MaarjAM reveals global and ecosystemic distribution patterns in arbuscular mycorrhizal fungi (Glomeromycota). New Phytol 188:223–241.

2. Maček I, Dumbrell AJ, Nelson M, Fitter AH, Vodnik D, Helgason T. 2011. Local adaptation to soil hypoxia determines the structure of an arbuscular mycorrhizal fungal community in roots from natural CO2 springs. Appl Environ Microbiol 77:4770–4777.

**FIG S2.** AM fungal community CCA (constrained correspondence analysis) plot for Slovenian meadow sites. The CCA axis one (CCA1) explained 43.66 % of AM fungal variability in AM fungal community composition, and CCA axis two (CCA2) 21.37 % of variability. All sampled community of AM fungi from Slovenian meadow sites are plotted separately. Significant (*P* < 0.001) environmental vectors are presented on CCA plot.

**FIG S3.** AM fungal community CCA (constrained correspondence analysis) plot for Slovenian meadow and forest sites, Italian and Czech sites. The CCA axis one explained 57.36 % of AM fungal variability in AM fungal community composition, and CCA axis two 21.50 % of variability. All sampled community of AM fungi from study sites are plotted separately. Significant (*P* < 0.01) environmental vectors are presented on CCA plot.

**FIG S4.** Rarefied accumulation curves of each sample from Slovenian meadow sites. Full line corresponds to 2010 samples, broken line to 2011 samples, with different colour of curves for each month sampled, red for April, green for June, blue for August and yellow for October sampling. MOTU represent sequences clustered at 97 % sequence similarity.

**Additional analyses using DESeq normalisation**

Compositional changes in Slovenian meadow AM fungal communities where data were normalised using DESeq were assessed using canonical correspondence analysis (CCA) which explained 47.90% of the variation within the taxa abundance data across the first two ordination axes (Fig. S5). Permutation test of CCA under a reduced model (10,000 permutations) was significant for Slovenian meadow sites (*P* < 0.005). AM fungal assemblages sampled from the mofette and control areas grouped into two distinct clusters on the first CCA ordination axis (ANOVA; *P* < 0.001), strongly correlated with CO_2_ and O_2_ concentration (r > 0.96; Fig S5). The environmental vectors used in the analysis were: soil factors (soil CO_2_ and O_2_ concentrations, pH, total nitrogen (N) and available phosphorus (P_2_O_5_)); climate variables (maximal and minimal temperature, sun hours and total rainfall in the 30 days prior to sampling) and seasonality (year and month), (Fig S5). The CO_2_ and O_2_ concentration are significantly (*P* < 0.001) and strongly positively (CO_2_) and negatively (O_2_) correlated with CCA axis one. Soil pH, available P_2_O_5_, year, and rain are significant (*P* < 0.01) but not aligned to either axis. Among the significant environmental vectors, the most variability was explained by concentration of CO_2_ (R^2^ = 93.16%), concentration of O_2_ (R^2^ = 86.95%), sampling years (R^2^ = 59.23%), pH (R^2^ = 54.13%), available P_2_O_5_ (R^2^ = 50.31%) and amount of rain (R^2^ = 32.42%) (Fig S5).

**
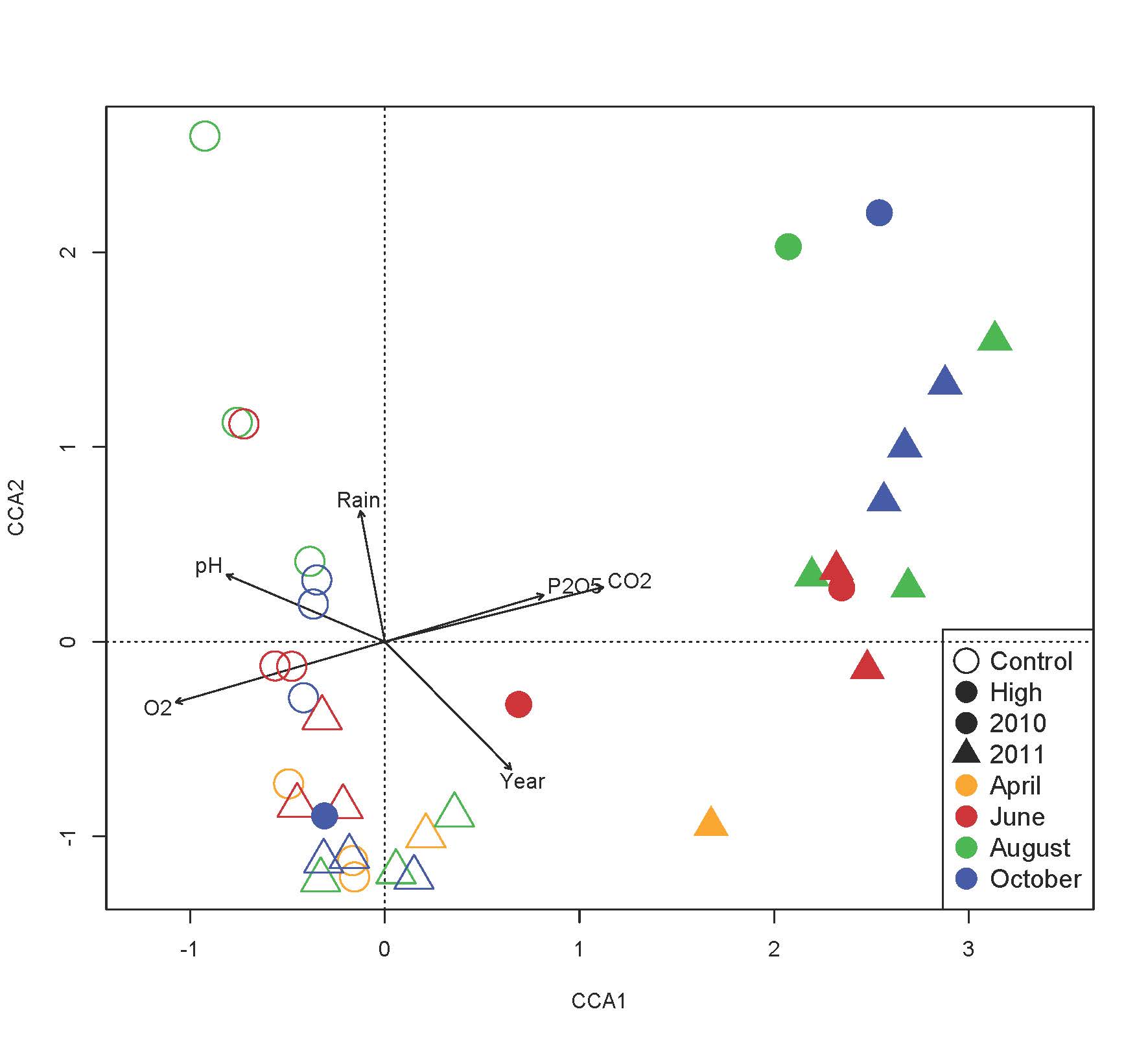
**

**Fig. S5**. AM fungal community CCA (constrained correspondence analysis) plot performed on data normalised using DESeq for the Slovenian meadow sites. The CCA axis one explained 30.86 % of AM fungal variability in AM fungal community composition, and CCA axis two 17.05 % of variability. All sampled community of AM fungi from Slovenian meadow sites are plotted separately. Significant (*P* < 0.01) environmental vectors are presented on CCA plot.

Significantly higher diversity of AM fungal communities was found in control areas compared to mofette areas sampled from Slovenian meadow sites when the data was normalised using DESeq (Fig. S6). We have observed significantly less MOTU rich, even and dominant AM fungal communities from mofette areas compared to those from control areas (three-way ANOVA with CO_2_ concentration, year and month as factors; *P* < 0.00001). There was no significant difference in mean alpha diversity of the AM fungal community when sampled in different years or in different months for each location within the study site.


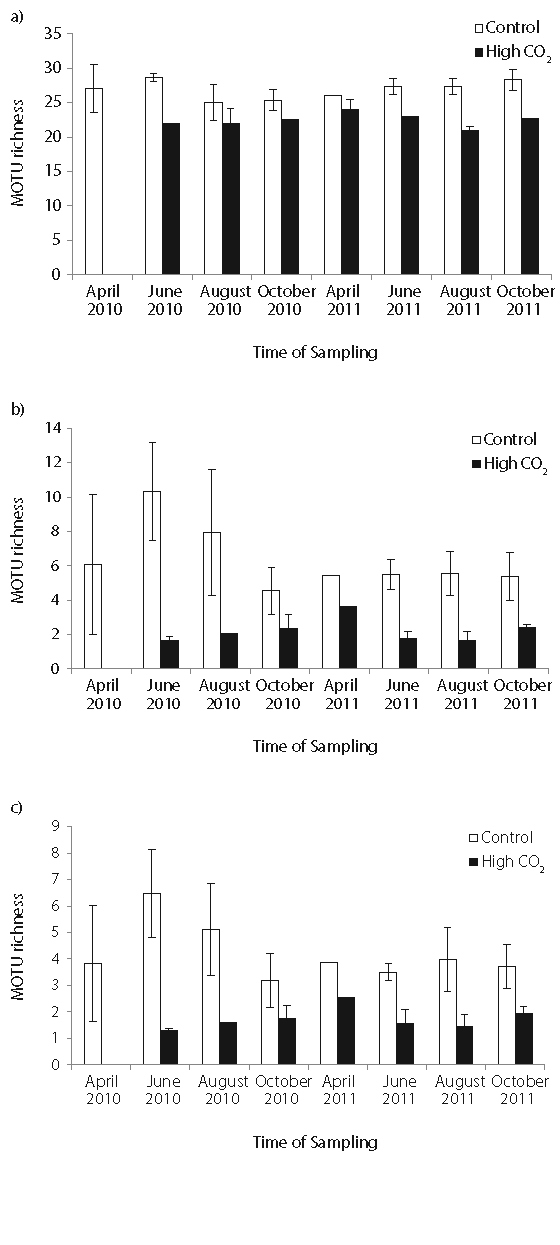


**Fig. S6.** Alpha diversity of AM fungi in the Slovenian meadow sites represented by MOTU richness, MOTU evenness and MOTU dominance (accessed by Hill’s numbers) on data normalised using DESeq. Three-way ANOVA showed significant differences between mofette and control areas (*P* < 0.00001), whereas no influence of sampling in different year or different months on diversity of AM fungi was observed.

In addition to Slovenian meadow sites, we have analysed data from Slovenian forest sites, Italian sites and Czech sites using canonical correspondence analysis (CCA) on data normalised using DESeq that revealed distinct separation of AM fungal communities from control and mofette areas from different study sites. For all sampled sites 73.14% of the variation within the species abundance data across the first two ordination axes was explained by the CCA (Figs S7, S8). Permutation test of CCA under a reduced model (10,000 permutations) was significant for all samples (*P* < 0.003). The environmental vectors used in the analysis were soil CO_2_ and O_2_ concentrations, pH, total nitrogen (N) and available phosphorus (P_2_O_5_). Among the significant environmental vectors, the most variability was explained by pH (R^2^ = 85.78%), total N (R^2^ = 59.94 %), available P_2_O_5_ (R^2^ = 59.56%), concentration of CO_2_ (R^2^ = 14.90%) and concentration of O_2_ (R^2^ = 12.28%) (Figs S7, S8).

**Fig. S7.** AM fungal community CCA (constrained correspondence analysis) plot performed on data normalised using DESeq for the Slovenian meadow and forest ites, Italian and Czech sites. The CCA axis one explained 50.74 % of AM fungal variability in AM fungal community composition, and CCA axis two 22.40 % of variability. All sampled community of AM fungi from study sites are plotted separately. Significant (*P* < 0.01) environmental vectors are presented on CCA plot.

**Fig. S8.** AM fungal community CCA (constrained correspondence analysis) plot performed on data normalised using DESeq for the Slovenian meadow and forest sites, Italian and Czech sites. The CCA axis one explained 50.74 % of AM fungal variability in AM fungal community composition, and CCA axis two 22.40 % of variability. In CCA plot, samples are pooled by year and location, and points are representing mean ± standard deviation of CCA scores for each axis. Significant (*P* < 0.01) environmental vectors are presented on CCA plot.

**
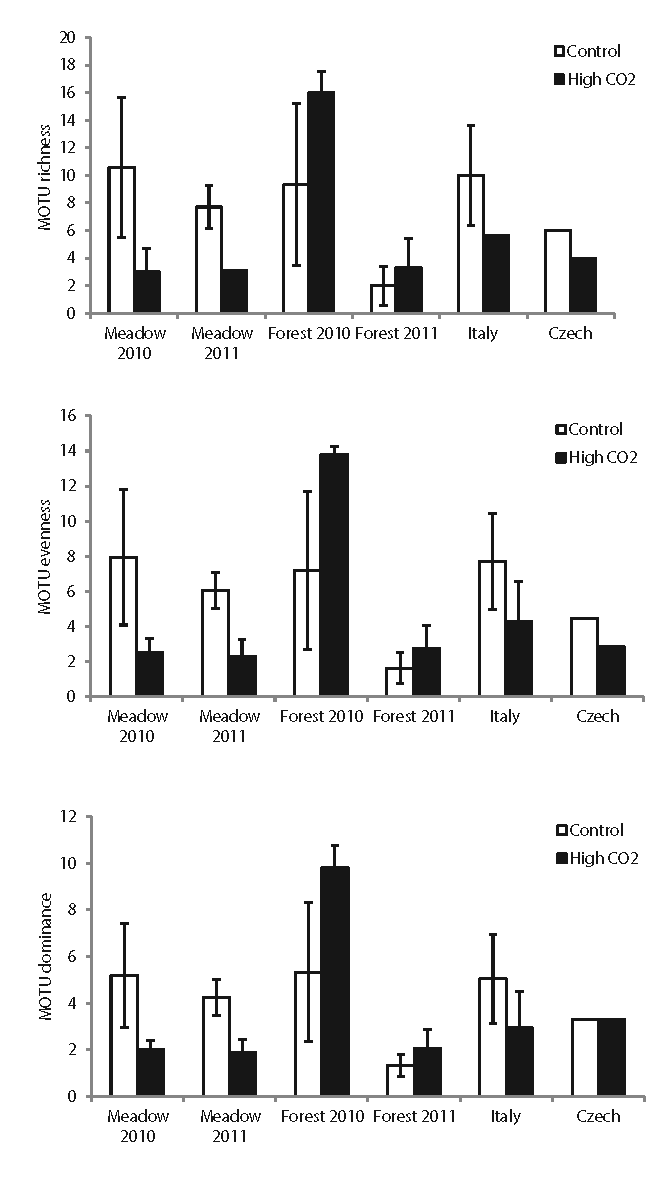
**

**Fig S9.** Alpha diversity of AM fungi sampled from Slovenian meadow and forest sites, Italian sites and Czech sites represented by MOTU richness, MOTU evenness and MOTU dominance (accessed by Hill’s numbers). Two-way ANOVA on dataset normalised using DeSeq showed significant differences between mofette and control areas (*P* < 0.00028) and among sites (*P* < 0.006), for all three indices.
